# Prediction of 492 human protein kinase substrate specificities

**DOI:** 10.1186/1477-5956-9-S1-S6

**Published:** 2011-10-14

**Authors:** Javad Safaei, Ján Maňuch, Arvind Gupta, Ladislav Stacho, Steven Pelech

**Affiliations:** 1 Department of Computer Science, University of British Columbia, Vancouver, Canada; 2 Department of Mathematics, Simon Fraser University, Burnaby, Canada; 3 Department of Medicine, University of British Columbia, Vancouver, Canada; 4 Kinexus Bioinformatics Corporation, Vancouver, Canada

## Abstract

**Background:**

Complex intracellular signaling networks monitor diverse environmental inputs to evoke appropriate and coordinated effector responses. Defective signal transduction underlies many pathologies, including cancer, diabetes, autoimmunity and about 400 other human diseases. Therefore, there is high impetus to define the composition and architecture of cellular communications networks in humans. The major components of intracellular signaling networks are protein kinases and protein phosphatases, which catalyze the reversible phosphorylation of proteins. Here, we have focused on identification of kinase-substrate interactions through prediction of the phosphorylation site specificity from knowledge of the primary amino acid sequence of the catalytic domain of each kinase.

**Results:**

The presented method predicts 488 different kinase catalytic domain substrate specificity matrices in 478 typical and 4 atypical human kinases that rely on both positive and negative determinants for scoring individual phosphosites for their suitability as kinase substrates. This represents a marked advancement over existing methods such as those used in NetPhorest (179 kinases in 76 groups) and NetworKIN (123 kinases), which consider only positive determinants for kinase substrate prediction. Comparison of our predicted matrices with experimentally-derived matrices from about 9,000 known kinase-phosphosite substrate pairs revealed a high degree of concordance with the established preferences of about 150 well studied protein kinases. Furthermore for many of the better known kinases, the predicted optimal phosphosite sequences were more accurate than the consensus phosphosite sequences inferred by simple alignment of the phosphosites of known kinase substrates.

**Conclusions:**

Application of this improved kinase substrate prediction algorithm to the primary structures of over 23, 000 proteins encoded by the human genome has permitted the identification of about 650, 000 putative phosphosites, which are posted on the open source PhosphoNET website (http://www.phosphonet.ca).

## Introduction

Integrated cell signaling pathways contribute to complex communications networks that govern basic and specialized cellular activities [2]. The ability of cells to perceive and correctly respond to their micro-environment is essential for growth, development, homeostasis, defense, and reproduction for tissue repair. Defective cell signaling, which can arise from gene mutation or toxic stimuli, has been linked to over 400 human diseases, including cancer, diabetes, autoimmunity, and neurological disorders [3]. Therefore, it is critical to map and track cell signaling networks with high precision in humans for diagnostic and therapeutic purposes. Protein phosphorylation catalyzed by protein kinases is the predominant mode of reversible post-translational control of proteins in eukaryotic cells.

Protein kinases transfer the gamma phosphate  of ATP to hydroxyl (-OH) groups found on amino acids in substrate proteins. Serine (S), threonine (T) and tyrosine (Y) represent the three amino acid residues most commonly targeted by these protein kinases [4-6]. Of the 23, 000 proteins encoded by the human genome, two-thirds have already been demonstrated to be phosphorylated at over 93,000 phosphosites [1]. Many of the targets of protein kinases include other protein kinases, and these enzymes can sequentially regulate each other in complex signaling networks. Our knowledge of the architecture of these kinase communications networks, which span from the cell plasma membrane to deep within the nucleus of cells, is very rudimentary. Most of the protein kinases are expressed in each cell in tens of thousands of copies, but a few are very restricted in their cellular expression patterns and have specialized functions. Under 10, 000 kinase–substrate phosphosite interacting pairs have been identified empirically, but probably over 10 million exist.

Domains are substrings of protein sequences that can evolve, function, and exist independently of the rest of the protein chain. The most common domain in protein kinases is the catalytic domain which carries out the actual phosphorylation of protein substrates. Most of the kinases feature a single highly related catalytic domain, some can have two of these catalytic domains, and few others have atypical catalytic domains. Throughout the catalytic domain of the kinases *specificity-determining residues* (SDRs) often directly interact with the side chains of amino acid sequences surrounding phosphosites (i.e. phosphosite region) in substrates [7]. Kinase-substrate binding conforms to a lock and key model, where a semi-linear phosphosite peptide sequence (surrounding the phosphosite) fits into a kinase active site that includes the SDRs.

Atypical kinases have completely different structures when compared to the typical protein kinases. They do not possess a catalytic domain similar to those found in typical kinases and appear to have evolved separately. No equivalent catalytic domain has been computed for them using alignment techniques. As a result, SDRs of the atypical kinases have to be searched through the whole surface of the protein, while for typical kinases they are contained within their catalytic domains. In this work, we have predicted the locations of SDRs in 488 human kinase catalytic domains and generated position-specific scoring matrices (PSSM) for each kinase.

The organization of the paper is as follows. In Section , we describe previous works related to the prediction of kinase phosphorylation specificities. In Section , kinase phosphorylation specificity is mathematically formalized. In Section , we propose our prediction algorithm for kinase phosphosite specificities based on consensus sequences of the phosphosite regions, and in Section , we improve the consensus idea by using profile matrices of each kinase, and finally in Section , we present our results.

## Related previous studies

There are many previous studies that aim to predict kinase specificities for protein substrate recognition and identify potential phospho-sites. The methods developed are usually based on computing consensus kinase recognition sequences, PSSM matrices or machine learning methods. Scansite [8], artificial neural networks [9] and support vector machines [10], conditional random fields [11], and voting based methods [12] are some of the examples of these approaches. A survey and comparison of the some of the mentioned prediction methods are represented in [13]. In addition, NetworKIN [6] and NetPhorest [14] are two significant efforts for modeling protein phosphorylation networks.

NetworKIN employs artificial neural networks and PSSMs to predict kinase domain specificities and uses protein-protein interaction databases such as STRING [15] to increase the accuracy of the prediction. Those kinases and substrates that are connected directly or indirectly (linked by a short path) in the STRING protein interaction database are better candidates to be selected in the phosphorylation network. NetworKIN covers only 123 kinases of the 516 known human protein kinases, since it does not compute phosphosite specificities for those kinases where there are no experimentally confirmed phosphosites. NetPhorest has slightly wider coverage with 179 kinases. Similar to NetworKIN, NetPhorest uses a combination of ANN and PSSM matrices for prediction, but it places related kinases within the same group (76 groups in total) and represents that all kinases in the same group have identical kinase phosphorylation specificities.

All the mentioned methods have two major problems: 1) they can only compute specificity of those kinases that are in the kinase–phosphosite pair databases; and 2) they are highly dependent on the number of confirmed phosphosites available for each kinase. The training data for all these works is usually retrieved from PhosphoSitePlus [16] and Phospho.ELM [17] which store information on kinase–phosphosite pairs. At this juncture, PhosphoSitePlus has gathered 95, 724 phosphorylation sites in 13,157 distinct proteins, while Phospho.ELM has 42,575 sites in 8,718 proteins. For less than 9500 of these phosphosites an upstream kinase is known.

## Kinase phosphosite specificity

Generally, there is a pattern in the phosphosite regions that a specific kinase phosphorylates. We shall refer to this pattern as its *kinase phosphosite specificity*. The reason is that each protein kinase has a unique 3D structure in its active site that is dictated by its own primary amino acid sequence, and only a small subset of peptide substrates would be expected to possess complementary structural conformations that can fully penetrate and fit into the kinase’s active site for phosphorylation. The amino acid sequences surrounding the phospho-acceptor residue in the best peptide substrates can often be aligned to generate a consensus sequence for optimal kinase recognition. Due to redundancies in the properties of various amino acid side chains, a series of kinase substrate analogs are feasible and these can best be represented in a kinase specificity matrix. These matrices display the observed or predicted frequencies of each of the 20 possible common amino acids at each position surrounding and including the phospho-acceptor amino acid residue. PSSM matrices and machine learning methods (e.g. ANN, HMM) can be used to generate a score for a given kinase and a substrate phosphosite region. Higher scores show that the kinase is more likely to phosphorylate that phosphosite. In other words, the score is a measure of kinase phosphosite specificity. To represent the pattern properly at least 9 amino acids (centered at phosphosite with four amino acids to right and left of the site) should be considered [13]. We decided to work with regions of length 15 because by considering six more amino acids we may obtain further information about the specificities for some kinases. Indeed, after computing the profile matrices of several hundred kinases we found that some additional information can be obtained from the added positions –7, –6, –5, +5, +6, and +7 (where 0 is the phosphosite, – means left and + means right of the phosphosite). However, increasing the length of the phosphosite regions to more than 15 may lead to the higher noise in the training data, which would make the prediction task harder.

We introduce a new PSSM matrix to predict kinase phosphosite specificities, which is computed in three steps described below.

### Profile matrix

We first compute the probability matrix, called the *profile matrix* for each kinase. Assume that it is experimentally known that kinase *k* phosphorylates *n* different phosphosite regions {*p*_1_, *p*_2_, … *p_n_*} of length 15. The profile matrix *P_k_* of kinase *k* is 21 × 15 matrix, where rows represent amino acids (including unknown amino acid ‘x’) and columns represent positions in the phosphosite regions. The reason of using symbol ‘x’ is that because in some positions of the primary structure of the proteins the exact amino acid is not known, and in addition to that some phosphosites are located close to the N-terminus (C-terminus) of the proteins and as a result no amino acid can be considered for the left (right) of the phosphosite region. In both of these cases we use symbol ‘x’ to create a consistent training set.

### Background frequencies of amino acids

Next, we compute the probabilities of each amino acid to appear on the surface of proteins. We call these probabilities *background frequencies of amino acids* and denote them by *B*(*i*), where 1 ≤ *i* ≤ 21. To compute background frequency we use all the 93, 000 confirmed phosphosite regions in human proteome. The reason is that all of these confirmed regions are on the surface of proteins, and hence, they can be a good sample of the protein surface. By examining the profile matrices of the kinases we have determined that positions –3, –2, 0, and +1 are particularly biased for kinase recognition, since all of them had a very low entropy. Therefore, we excluded these positions for the computation of the background frequency of each amino acid.

### PSSM matrix

Now having a profile matrix of each kinase and the background frequency of amino acids, the PSSM matrix for kinase *k* is typically computed using log odds ratio measure:(1)

where 1 ≤ *i* ≤ 21 and 1 ≤ *j* ≤ 15. The problem with this method is that since the profile matrix *P_k_* computed using experimental data contains many zeros, the resulting PSSM matrix *M_k_* has many –∞ values, and consequently, *M_k_* is not smooth enough for the prediction. Various smoothing techniques [18] are applied here to avoid zeros and –*∞* values, but we use a different approach which produces better PSSM matrices for prediction:(2)

where the exponent 1*.*2 was determined experimentally to achieve the best results.

The logic behind this method is similar to log odds ratio. If the probability of amino acid *i* at position *j* of profile matrix is bigger than the background frequency of *i* then that amino acid is a positive determinant, while if it is less than the background frequency it is a negative determinant for the phosphosite region containing *i* at position *j* to be recognized by that specific kinase. For a given candidate phosphosite region we are interested to see more positive and less negative determinant amino acids to predict it as a phosphosite.

### Score of phosphosite region

Having PSSM matrix *M_k_* for kinase *k*, we can compute how likely a given candidate phosphosite region *r* = *r*_1_*r*_2_…*r*_15_ is going to be phosphorylated by kinase *k*. This value is called *kinase specificity score S* and is computed as follows.(3)

## Prediction of PSSM for kinases without substrate data

**Figure 1:**
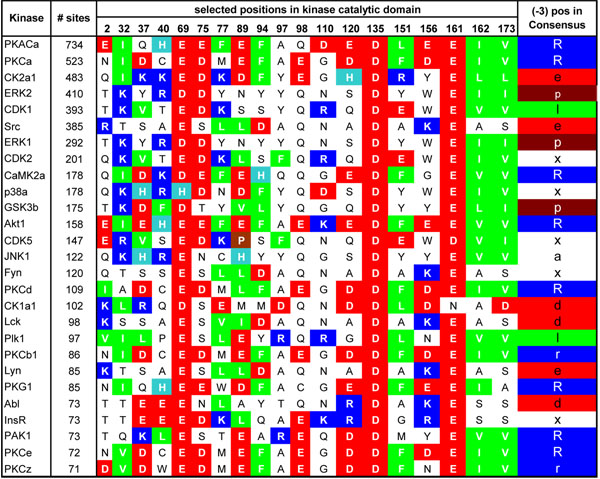
**Kinase catalytic domain alignment.** Some of the well characterized protein kinases with critical amino acids in their catalytic domains. In the right most column, (–3) position of the consensus sequence of each kinase is shown. Strongly positively charged amino acids (R, K) are represented as blue, weakly positively charged histidine as light blue, strongly negatively charged amino acids (E, D) as red, hydrophobic amino acids (L, V, I, F) as green, and proline (P) as brown.

**Figure 2:**
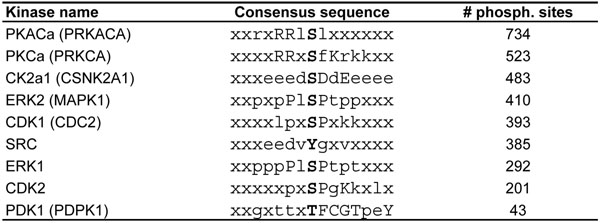
**Kinase consensus sequences.** Consensus sequences of some of the kinases for which we have the most number of experimentally confirmed phosphorylation sites in protein substrates (except for PDK1, which is shown because it is a threonine-specific protein kinase). Phosphorylation sites are marked by bold font at the center of consensus sequence. Number of total phosphorylation sites for each kinase is shown in the last column.

In this section, we present our algorithm for prediction of PSSM matrices based on their catalytic domains. The idea is that those catalytic domains in different kinases which have similar SDRs tend to have similar patterns in the phosphosite regions. To quantify the similarity of catalytic domains of kinases we perform multiple sequence alignment (MSA) of catalytic domains using ClustalW algorithm [19]. The result of the MSA is not quite accurate as it has many gaps, therefore, the alignments were manually modified. We perform this alignment on 488 catalytic domains of the typical protein kinases. The length of each kinase catalytic domain after MSA is 247. For 224 domains in the alignment we compute *consensus sequences* using 6, 515 confirmed kinase–phosphosite pairs. Figure 1 represents portions of the catalytic domain after MSA of some of the best characterized kinases for which the most phosphosites have been identified. To generate the consensus sequence of each kinase, profile matrix of each kinase is computed using the confirmed phosphosite regions of each kinase. For each position in the consensus sequence the amino acids with the maximum probability in that position is selected. If the probability is bigger than 15% then a capital letter is used to represent that amino acid, if it is less than 15% and bigger than 8%, a small letter is used, and if it is less than 8%, symbol ‘x’ is used in that position of the consensus sequence. ‘x’ here is a ”don’t care” letter and it means that any amino acid can appear in that position of the phosphosite region of a kinase. Therefore, those kinases that have more ‘x’ in their consensus sequence are more general and can phosphorylate more sites than the others. In Figure 2 consensus sequences of some of the well studied kinases are presented.

In what follows we use the example in Figure 1 to explain how mutual information and charge information are applied to find SDRs on the catalytic domains of the kinases.

### Mutual information

Each position in catalytic domains or consensus sequences can be considered as a random variable which can take 21 different values. Both random variables can take any of the 20 amino acids. In addition, the random variables in domains can also take the gap value ~, while the random variables in consensus sequence can take the unknown value ‘x’. In information theory the mutual information of two random variables is a quantity that measures the mutual dependence of the two variables [20]. We can use this measure here to find out which two positions in consensus and catalytic domain are highly correlated. Formally, the mutual information of two discrete random variables *X* and *Y* is defined as:

where *p*(*x*, *y*) = **P**(*X* = *x*, *Y* = *y*), *p*_1_(*x*) = **P**(*X* = *x*), and *p*_2_(*y*) = **P**(*Y* = *y*). The higher mutual information, the more the random variables are correlated. In our context, *X* is a position in the kinase catalytic domain, *Y* is a position in the consensus sequence, *A* is a set of amino acids plus *~* and *B* is a set of amino acids plus ‘x’.

### Charge information

**Figure 3:**
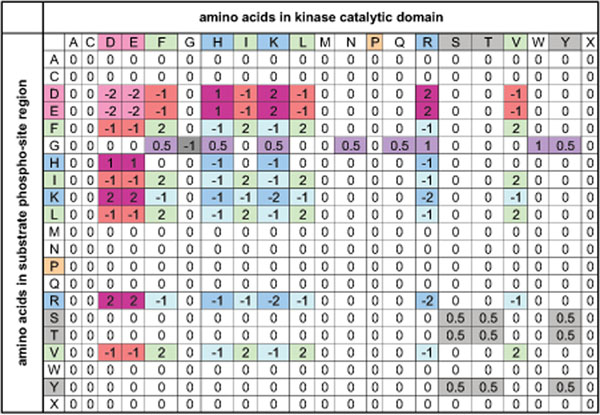
**Residue interaction matrix.** Residue interaction matrix *R*. Rows show the amino acids in the phosphosite regions and columns are amino acids in the catalytic domain of the kinases. Negatively charged amino acids (D, E) are red, positively charged amino acids (K, R, H) are blue, hydrophobic amino acids (F, I, L, V) green, proline as orange, and phosphorylation site residues (S, T, Y) are represented as gray. ‘x’ also corresponds to the absence of an amino acid, which occurs for phosphosites located at the N-and C-termini of proteins. This figure was derived from knowledge of the structure and charge of the amino acid side chains.

Negatively charged amino acids interact with positively charged, and hydrophobic amino acids with hydrophobic ones. Therefore, if a position in the catalytic domains (see Figure 1) tends to have many negatively charged amino acids and a position in the consensus sequences tends to have more positively charged amino acids, it is likely that these two positions are interacting with each other. Therefore, we define *charge dependency C*(*X*, *Y*) of two positions (random variables), one in kinase catalytic domains (*X*) and the other in consensus sequences (*Y* ), as follows.(4)

where *n* is the number of kinases with consensus pairs (in our case 224). *R* is also residue interaction score of two different amino acids, c.f. Figure 3, *x_k_* is the amino acid of the *k*^th^ kinase at position *X* of the catalytic domain and *y_k_* is the amino acid of the corresponding consensus sequence at position *Y*.

Residue interaction matrix shown in Figure 3 estimates the strength of a bond created between amino acids in the average case independent of their distance. Negatively (positively) charged amino acids repel themselves (score –2 in the interaction matrix *R*) and they attract positively (negatively) charged amino acids (score +2). Histidine (H) has a smaller positive charge than lysine (K) and arginine (R). Therefore, scores for it are +1 for interacting with negatively charged amino acids and –1 for interacting with positively charged amino acids. Hydrophobic amino acids attract each other (score +2) while they repel both positively and negatively charged amino acids (score –1). S, T and Y residues have a weak tendency to bind to each other (score +0*.*5), while they are completely neutral with the other amino acids (score 0). For all the amino acids discussed so far, it is not relevant whether they are in the kinase catalytic domain or phosphosite region. In both situations the score is the same, which makes the interaction matrix symmetric. However, glycine (G) is favored to be in the phosphosite region, because it is a small amino acid that creates a pocket on the surface of the region that permits the catalytic domain of the kinase come closer to the region. The reason that we do not consider effect of G in the catalytic domain is that we are unclear about the 3D structure of the most kinase catalytic domains, while phosphosite regions are linear or semi-linear.

If we look at Figure 1 we observe that columns 69, 135, and 161 are quite conserved with negatively charged amino acids. Since at (–3) position of the consensus sequences of the substrates mostly positively charged amino acids (e.g. arginine (R)) appear, these positions have a high charge dependency score *C* and are strong candidate positions for interaction with (–3) position of the phosphosite regions. On the other hand, these positions are very conserved and they seem to be uncorrelated with the (–3) position of the phosphosite regions (e.g. when the (–3) position is positively charged or neutral, position 69, 135, and 161 are still negatively charged). Therefore, we need a criterion to combine the correlation and charge dependency measures. The following equation combines these two measures.(5)

where *C_c_*(*X*, *Y* ) is called *correlation–charge dependency* of two positions *X* in catalytic domains and *Y* in consensus sequences.

Using this hybrid criterion *C_c_*(*X*, *Y* ) in our example, column 120 gets the maximum correlation charge dependency in Figure 2. It is usually preferred that for a particular position in consensus sequences, SDRs in catalytic domain stay near each other, because they can easily interact with that position in consensus sequences. For example, positions 120 and 121 should be preferred to positions 120 and 220. However, in the 3D structure of the protein kinase domain, amino acids that are well separated in the sequence could be situated next to each other. In view of such exceptions, we did not include this preference in our model. Algorithm 1 computes the best SDRs (positions *X* in the catalytic domain) for each kinase consensus sequence position *Y* and their interaction probabilities  using correlation–charge dependencies.

By examination of the x-ray crystallographic 3D structures of 11 protein kinases co-crystallized with peptide substrates, we determined that usually at most seven SDRs may interact with an amino acid position on the substrate phosphosite region, therefore we set the value *m* in Algorithm 1 to 7. Furthermore, considering higher values for *m* will result in very smooth specificity matrices more or less similar for all the kinases.

From 516 known human protein kinases, 478 kinases are typical kinases with 488 known catalytic domains and the remaining 38 kinases are all atypical kinases and we have phosphosite specificity data only for four of them. Algorithm 2 computes the profile and PSSM matrices for 488 catalytic domains, using the SDRs determined by Algorithm 1. The formula in Line 5 of the Algorithm 2 is based on the observation that those interactions which have higher correlation–charge dependency are more important in estimation of profile matrices.

## Using profile matrices for prediction

**Figure 4:**
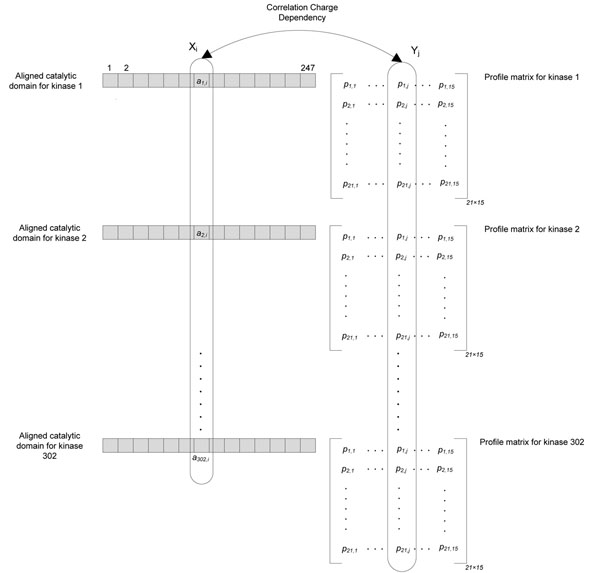
**Computing correlation charge dependency in profile matrix based module of the predictor.** In the left part of the figure the aligned catalytic domains of the 302 training kinases is shown, and on the right hand side for each kinase the profile matrix is drawn. It is clear that the same columns in all the kinase profile matrices create only one random variable, where its correlation to the aligned catalytic domain should be studied.

In Section we used phospho-peptide consensus sequences of each kinase to compute correlation charge dependency and SDRs, because it was easier to describe. Another idea was to use profile matrices of each

kinase in Algorithm 1 without determination of their consensus sequence. With this strategy we use more information and it it might allow for better prediction, while on the other hand it may lead to overfitted results. In Section , we will test both of these algorithms (1. consensus based and 2. profile based), and compare the results. In profile matrix based method, the main difficulty is that for the random variable *Y_j_* (column *j* in the aligned consensus sequences) we do not have the correlated values of the random variable *X_i_* (column *i* in the aligned catalytic domains). Instead, for each value in *X_i_* we have 21 different amino acid probabilities of *Y_j_.* Assume *a_k_*_,_*_i_* is the amino acid in the aligned catalytic domain of the kinase *k*, also let be the probability of the *l^th^* amino acid (1 ≤ *l* ≤ 21) at position *j* (1 ≤ *j* ≤ 15) of the profile matrix of kinase *k.* Figure 4 represents these notations in a visual manner. Before computing the charge dependency correlation of two columns (or random variables) *X_i_* and *Y_i_* we compute the probability of amino acids in each random variable. **P**(*X_i_* = *x*) is computed by maximum likelihood estimation using *a_k_*_,_*_i_* amino acids as follows:(6)

where 〈*x* = *a_k_*_,_*_i_*〉 is the indicator variable taking values ones (when *a_k_*_,_*_i_* is equal to *x*) and zeros otherwise, *w* is the number of all kinase catalytic domains. **P**(*Y_j_* = *l*) or **P**(*Y_j_* = *y*) is also computed by the following equation:(7)

Similar to the previous section we replace **P**(*X_i_* = *x*) and **P**(*Y_j_* = *y*) with *p*_1_(*x*) and *p*_2_(*y*) respectively. *p*(*x*, *y*) is also computed using maximum likelihood estimation as follows:(8)

having *p*_1_(*x*), *p*_2_(*y*), and *p*(*x*, *y*) we can now compute the charge correlation dependency using Equation (5) and pick top values for SDRs.

Another modification which should be applied on the consensus method is to change conditional probability of phospho-peptide positions given SDRs (which is shown by **P**(*Y_j_*|*Z_j_*_,_*_l_*) ) in Line 5 of Algorithm 2. This probability according to Bayes’ theorem equals to  and both numerator and denominator can be computed similar to Equations (8) and (6) respectively.

## Results and discussion

In this study we perform four different computational simulations on the proposed predictor. The first simulation evaluates the accuracy of the predicted profile matrices by consensus and profile-based modules of the predictor. The second simulation is to build PSSM based on the predicted profile matrices and use them as classifiers for each kinase, and to compute the confusion matrices to further determine the accuracy of the predictor. The third simulation is to compare NetPhorest with our predictor based on our set of kinase-phosphosite pairs. Finally, the last simulation is to compare NetPhorest and our method with NetPhorest data sets. Each of these tests are explained more thoroughly in the following subsections.

### Comparison of profile matrices

In this simulation we compare the accuracy of the predicted matrices with the original matrices computed based on experimental kinase-phosphosite pairs. For 308 kinases we could gather 9,012 confirmed phosphosites from PhosphoSitePlus, Phospho.ELM, the scientific literature and other databases. The confirmed kinase-phosphosite pairs were partitioned into two training and test sets. The test set contains top five kinases that have the most phosphosite data. The reason is that by choosing those kinases in the test set we will be almost confident that the experimentally computed profile matrices are correct and reasonable to compare with the predicted matrices. The training set contains 302 kinases with 6, 515 experimentally confirmed phospho-peptides. To start running our predictor on the training data we needed to generate reliable consensus sequences (for the consensus based module of the predictor) of phospho-peptide for each kinase first, therefore we eliminated those kinases having less than 10 phospho-peptides.

Among the 302 kinases in the training set, 224 kinases had more than 10 phospho-peptides and we could compute 224 consensus sequences for each using the process explained in Section . From about 516 human kinases, we gathered 488 catalytic domains in 478 human typical protein kinases, aligned these catalytic domains, and used Algorithms 1 and 2 to compute SDRs and profile matrices.

**Figure 5:**
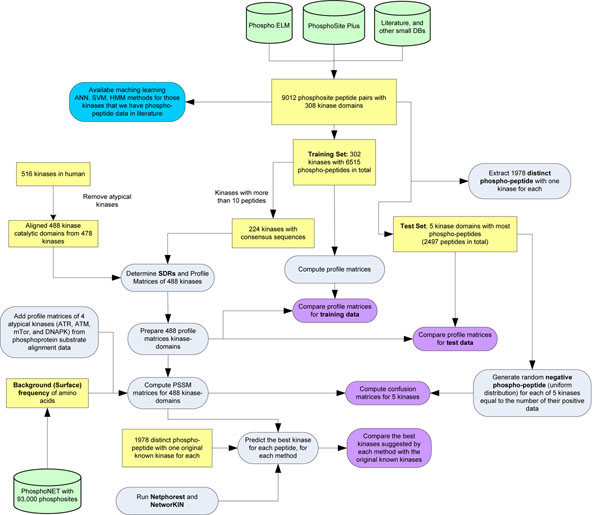
**Data and process flow of the experiments.** The figure shows the order of creating the datasets for the computational simulations and comparison of our predictor results with current state of the art methods such as NetPhorest and NetworKIN.

**Figure 6:**
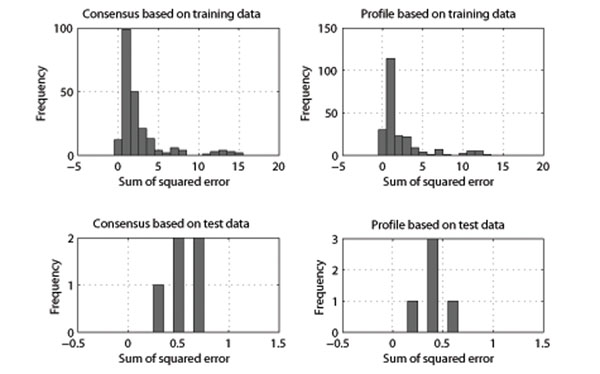
**Comparison of predicted vs. experimentally computed profile matrices.** The figure contains four different histograms, where each diagram represent the sum of squared error of the predicted profile matrices and experimentally computed profile matrices based on confirmed phospho-peptide pairs for each kinase. x-axis is the sum of squared error, and y-axis is the frequency or number of matrices which have the specified error. Left histograms are based on consensus based module of the predictor and right histograms related to the profile matrix based module of the predictor. Top histograms show the training set, and the bottom histograms are the results on the five test kinases. Total sum of squared error (SSE) for the consensus based module on training data is 584*.*89, and on the test set is 2*.*66, while total SSE for the profile matrix based on training data is 494*.*22, and on the test set is 1*.*99.

To evaluate these predicted matrices, we also computed the profile matrices of 302 kinases in the training set computed by the method described in Section (empirical matrices), and the results were compared using sum of squared differences. Figure 5 illustrates how we set up this comparison, and Figure 6 shows the distribution of these errors. This figure presents the results for the profile matrix based module of the predictor as well. It is evident that the majority of the predicted matrices are extremely similar to those generated by known substrate alignments. Interestingly, the results on the test set are more accurate (with sum of squared error less than 1) than the predicted results on the training set (which can be up to 10 to 15) for both modules. The reason is that for each kinase in the test set there are more experimental substrate peptides, and as a result empirically computed matrices are closer to the correct specificity of each kinase. However, in the training set there are many kinases with less than 20 – 30 experimentally confirmed substrate peptides and we may expect their empirically computed matrices are not close the correct profile of each kinase. The profile matrix based module used more information than the consensus module, and not only does it not overfit on the data, but also it has more accuracy with both test (with a total of 1*.*99 sum of squared error (SSE) for all five kinases) and training set (with a total of 494*.*22 SSE). As evident in Figure 6, the consensus based method had higher errors with a total of 584*.*22 SSE for all kinases in the training set and a total of 2*.*66 SSE for all kinases in the test set.

### Computation of confusion matrices and accuracy

**Figure 7:**
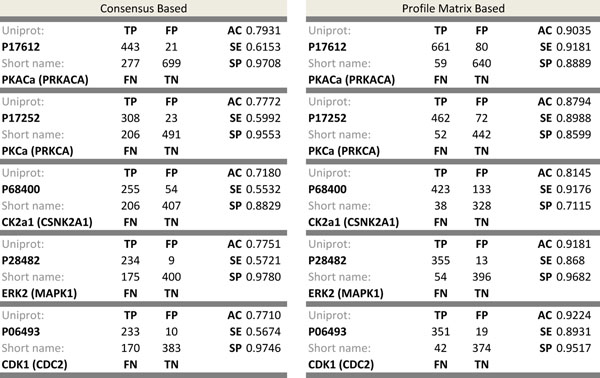
**Confusion matrices for two modules.** The figure includes two tables and each table represents the classification power or consensus module (on the left) and profile matrix module of the predictor (on the right). In each table confusion matrix is represented by true positive (TP), false positive (FP), false negative (FN), and true negative (TN). Also accuracy (AC), sensitivity (SE), and specificity (SP) metrics are computed based upon the confusion matrices.

In this simulation, we measured the accuracy of each predicted kinase substrate specificity for those kinases in the test set. For this, we determined classifiers for each kinase and prepared positive and negative instances for each classifier. We used PSSM matrices of each kinase as a classifier and took the confirmed substrate peptides of each kinase in the test set as positive instances. For negative instances, unlike previous attempts [10,11], we randomly generated negative instances for each kinase in the test set equal to the number of its positive instances using a uniform distribution. The reason for this is that if we choose those substrate peptides that are not experimentally confirmed but are in the substrate protein as negative instances, it is probable that in future studies (e.g. From mass spectrometry analyses) they may later prove to be positive instances. Afterward, for any given substrate phospho-peptide, we computed the score of the PSSM matrix as in Equation (3) and if the score was less than zero it was declared that the substrate phospho-peptide was negative for the kinase in question. Otherwise, we accepted the given substrate phospho-peptide as a candidate peptide phosphorylatable by the kinase. Figure 5 is also helpful for showing the flow of the data for preparing positive and negative substrate phospho-peptides for the top five test kinases. For all the kinases in the test set similar results were computed, and the classifiers were successful in identifying most of the negative instances (low rate of false positives), but they were apparently much less efficient for identifying all the positive instances (high rate of false negatives). Approximately 77% accuracy, 60% sensitivity and 95% specificity values were computed for all the classifiers in the test set. Figure 7 represents the exact confusion matrix, accuracy, sensitivity and specificity values for each kinase in the test set for both the consensus and profile matrix based sub-modules of our predictor. It was observed that the sensitivity for all classifiers in the consensus based method was low, while this disadvantage was eliminated in the profile matrix based method with 10% higher accuracy compared to the consensus method.

### NetPhorest vs. our predictor

In this part we compare the accuracy of NetPhorest and our method on the same kinase– phosphosite pairs. To fulfill this task we extracted 1, 978 distinct phospho-peptide–kinase pairs from the total 9, 012 pairs discussed at the beginning of this section. This set was used afterward, for measuring the accuracy of each predictor. For each phospho-peptide in this set we stored the best kinase (highest score) suggested by NetPhorest and our method. Afterward, we measured how many of the predicted kinases are matching with the original kinases in the 1978 pairs. Because NetPhorest works based on kinase groups and predicts the best kinase group for the input phospho-peptide, anytime that the original kinase falls into the predicted kinase group we consider it as matching. For instance, if the input pair is <TRKLMEFpSEHCAIIL, TGFbR2> and NetPhorest predicts kinase group ACTR2_ACTR2B_TGFbR2_group for the input phospho-peptide ‘TRKLMEFpSEHCAIIL’ we accept it as a hit. After running this experiment, we observed that NetPhorest was successful in 72 of the pairs and our proposed method in 82 cases. By this experiment we show that our method outperformed NetPhorest on three accounts: Firstly, it has a higher rate of matches with empirical data. Secondly, it separately considers 492 different kinase catalytic domains, whereas NetPhorest matchings are based on 76 groups of related kinases. Thirdly, 2497 training phospho-peptide pairs for five well studied kinases PKACa, PKCa, CK2a1, ERK2 and CDK1 are not used in training the classifiers and they are solely kept for testing the algorithm, while NetPhorest uses most of these data to improve its accuracy.

### NetPhorest vs. our predictor based on NetPhorest confirmed sites

**Figure 8:**
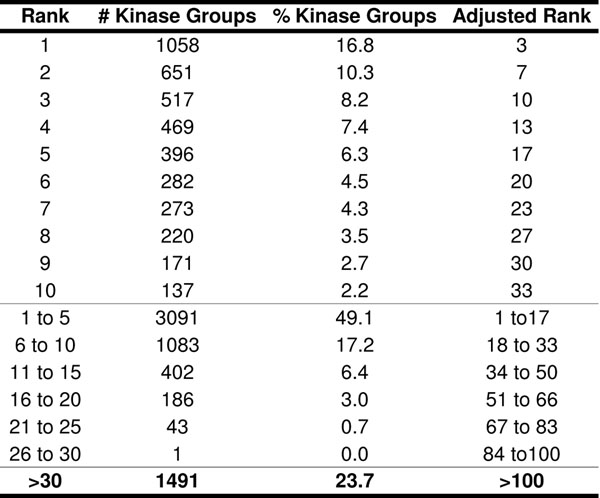
**Comparison with NetPhorest predictions.** This table shows how many times the NetPhorest kinases groups fall to the ranking groups 1 to 30 as determined in our kinase substrate predictor algorithm. For instance the first row illustrates that 1058 NetPhorest kinase groups (16*.*8%) were similarly predicted by our algorithm as the best kinase groups for the specific phospho-peptides. Because every kinase group in NetPhorest contains 3*.*3 kinases in average, the rank can be adjusted and we can say 1058 NetPhorest kinases (and not kinase groups) were similarly predicted by our algorithm as the best three kinases for the specific phospho-peptides.

In this part we compare consensus module of the predictor with NetPhorest based on confirmed phospho-peptides existing in NetPhorest database. At this juncture, NetPhorest contains 10, 261 confirmed phosphosites and has 76 specified groups for a total of 179 kinases linked to phosphorylation of 8, 746 of those sites. In this dataset, some phosphosites had more than one kinase phosphorylating them. To compare our predictor with NetPhorest easier we retained only the best kinase for each phosphosite. We also considered only those kinases with our predictor algorithm that were included in the list of 179 protein kinases covered by NetPhorest. As a result, the number of kinase–phosphosite pairs was reduced to 6, 299. To examine how many of these kinase-phosphosite pairs were consistent with our predictor, we subjected these 6, 299 phosphosites to our predictor algorithm to determine which individual kinases were more likely to phosphorylate these sites. We ranked the 179 protein kinases based on their calculated PSSM scores for each NetPhorest confirmed phospho-site region. It was desirable that the experimentally confirmed kinases for each phosphosite region had high PSSM scores in our predictor. However, we cannot expect these confirmed kinases always have maximum PSSM scores, because although these kinases were experimentally demonstrated to phosphorylate those phosphosites, it is unclear that they are always the best possible matches. Figure 8 shows that 1058 NetPhorest kinase groups were similarly predicted by our algorithm as the best kinase groups for the specific phospho-peptides, and 651 kinase groups were predicted as the second best kinase groups, etc. On average each NetPhorest kinase family has 3*.*3 kinases and because our algorithm works based on individual kinases and not a group, we adjusted the ranks and intervals for the results from our algorithm accordingly to provide direct comparison. It is evident that 35 percent of the NetPhorest predicted kinases groups corresponded to the top 10 candidate kinases proposed by our algorithm. Therefore, our predictor had similar prediction accuracy to NetPhorest, but we achieved coverage with three times as many different protein kinases and with individual assignments rather than groups of kinases. This result is also shown in our previous work in BIBM 2010 [21].

## Competing interests

The authors declare that they have no competing interests.
